# A GWAS in uveal melanoma identifies risk polymorphisms in the *CLPTM1L* locus

**DOI:** 10.1038/s41525-017-0008-5

**Published:** 2017-03-10

**Authors:** Lenha Mobuchon, Aude Battistella, Claire Bardel, Ghislaine Scelo, Alexia Renoud, Alexandre Houy, Nathalie Cassoux, Maud Milder, Géraldine Cancel-Tassin, Olivier Cussenot, Olivier Delattre, Céline Besse, Anne Boland, Jean-François Deleuze, David G. Cox, Marc-Henri Stern

**Affiliations:** 1 Inserm U830 and Ensemble Hospitalier, PSL Research University, Institut Curie, Paris, France; 2 UMR5558, Laboratoire de Biométrie et Biologie Evolutive, Equipe Biostatistique-Santé, Université Claude Bernard-Lyon 1, Lyon, France; 3 Service de Biostatistique-bioinformatique, Hospices Civils de Lyon, Lyon, France; 4 International Agency for Research on Cancer (IARC), Lyon, France; 5 INSERM U1052, CNRS UMR5286, Université Lyon 1, Centre de Recherche en Cancérologie de Lyon, Lyon, France; 6 UPMC University Paris 06 GRC n°5, CeRePP, Hôpital Tenon, Paris, France; 7 Centre National de Génotypage, Institut de Génomique, CEA, Evry, France

## Abstract

Uveal melanoma, a rare malignant tumor of the eye, is predominantly observed in populations of European ancestry. A genome-wide association study of 259 uveal melanoma patients compared to 401 controls all of European ancestry revealed a candidate locus at chromosome 5p15.33 (region rs421284: OR = 1.7, CI 1.43–2.05). This locus was replicated in an independent set of 276 cases and 184 controls. In addition, risk variants from this region were positively associated with higher expression of *CLPTM1L*. In conclusion, the *CLPTM1L* region contains risk alleles for uveal melanoma susceptibility, suggesting that *CLPTM1L* could play a role in uveal melanoma oncogenesis.

## Cancer: Risk allele identified for melanoma of the eye

Researchers have discovered an important genetic risk variant linked to uveal melanoma, a rare malignant tumor of the eye. Marc-Henri Stern from Institut Curie in Paris, France, and colleagues compared more than 860,000 single DNA variants covering the entire genome, from the genomes of 259 people with uveal melanoma and 401 healthy controls, all of whom were of European ancestry. The researchers found that a series of closely linked gene variants on the short arm of chromosome 5 were significantly more common in the melanoma patients. They confirmed the association between this genomic region and disease risk in an independent cohort of 276 cancer cases and 184 controls. Expression analyses showed that the *CLPTM1L* gene contained in this region was more expressed in people with the risk variants, pointing to *CLPTM1L* playing a role in tumor development.

## Introduction

Uveal melanomas (UM) arise from melanocytes in the uveal tract, including the choroid, ciliary body and iris. Choroidal uveal melanoma are the most common form of UM and of intraocular primary tumor in adults. UM represent about 4–5% of all melanomas and has an incidence rate of 5.6 cases per million person-years (~500 new cases a year in France). Prognosis is dismal when the disease spreads, frequently to the liver.

Despite their common neural-crest lineage, uveal and cutaneous melanocytes arise for cranial and trunk neural crests, respectively. This may contribute to the major differences between uveal and cutaneous melanoma in terms of epidemiology, genetics, mechanisms of malignant transformation and clinical outcome. Most UM cases are associated with two main genetic events. The first event includes mutually exclusive activating mutations leading to the constitutive activation of the Gαq pathway targeting most often *GNA11* or *GNAQ* genes, encoding G-alpha proteins, or more rarely of CYSLTR2, a GPCR coupled with Gαq, or of PLCB4, downstream of Gαq.^1, 2^ The second genetic event includes recurrent mutations targeting the *BAP1*, *SF3B1* and *EIF1AX* genes in an almost mutually exclusive manner, with *BAP1* inactivation associated with a high risk of metastasis.^3–6^

Epidemiological studies have shown that UM affects mainly populations of European ancestry, with few cases in African–Americans and Asian populations. The Surveillance, Epidemiology, and End Results (SEER) database recently showed that 97.8% of UM cases in the United States occur in the population of European ancestry,^7, 8^ with incidence rate ratios of 0.05 and 0.07 for individuals of African–American and Asian/Pacific Islander origins, respectively.^9^ Furthermore, meta-analyses revealed that fair skin and blue/gray eyes are significantly associated with UM.^10^ This bias for European origin and fair skin is reminiscent of epidemiological data from cutaneous melanomas and suggests a role of pigmentation protecting against ultraviolet (UV) exposure in the pathogenesis of UM. In accordance, a recent association study demonstrated that pigmentation traits are risk loci for UM.^11^ However, while the incidence of cutaneous melanoma has increased in Europe, North America and Australia, incidence of UM has remained stable.^12^ Furthermore, whole genome sequencing of UM tumors demonstrated the absence of UV mutational signature in this disease.^4^ An alternative hypothesis to skin pigmentation that may explain the lower incidence of UM in Afro–American and Asian populations is the prevalence of inversely associated alleles in these populations, or conversely the prevalence of risk alleles in populations of European ancestry, as shown for Ewing sarcoma, a disease which is also mostly found in populations of European ancestry.^13^

## Results

### Association analysis

**Fig. 1:**
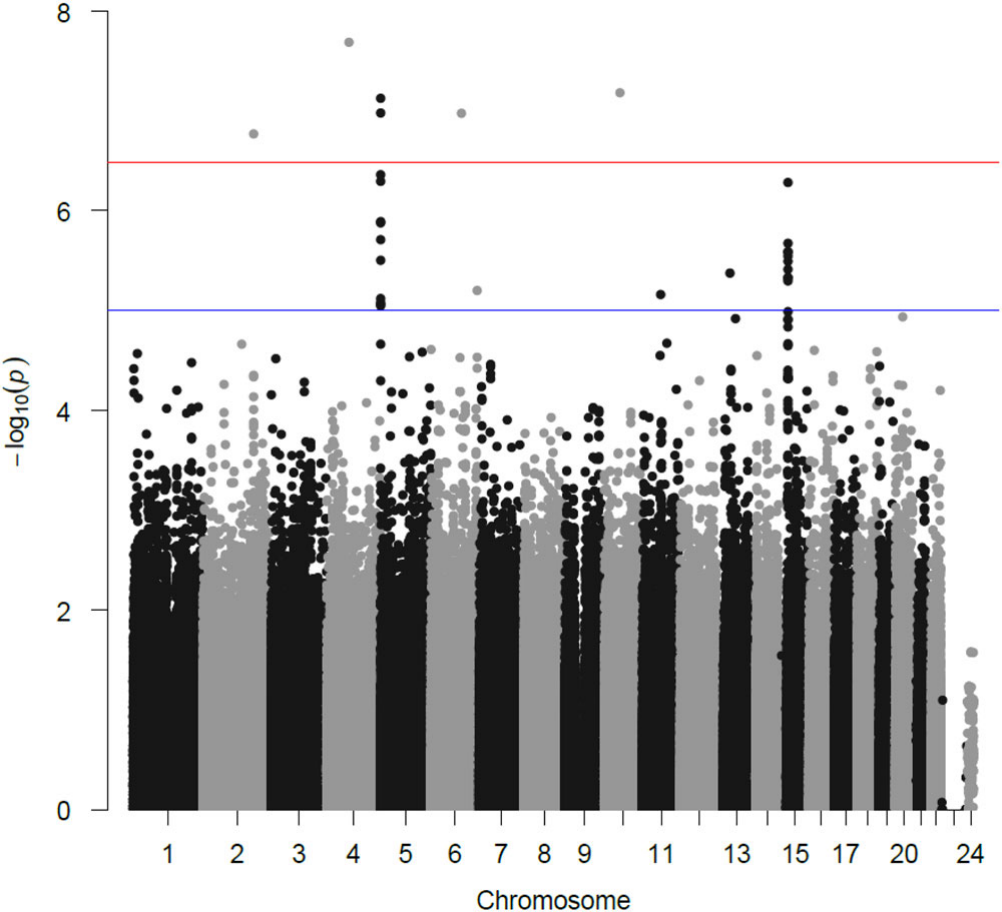
Manhattan plot for the discovery series (259 UMs and 401 CTLs). The log_10_ of the association test *P*-value of 866,782 SNPs is plotted against its physical chromosomal position. Chromosomes are shown in alternate *black* and *grey*. SNPs above the *red line* represent those with a *P-*value <3.3 × 10^−7^ and were considered as significantly associated with uveal melanoma risk. The *blue line* represents the suggestive line (*P*-value <1 × 10^−5^). Significance was measured using unconditional logistic regressions and the Cochran–Armitage test for trend

**Fig. 2:**
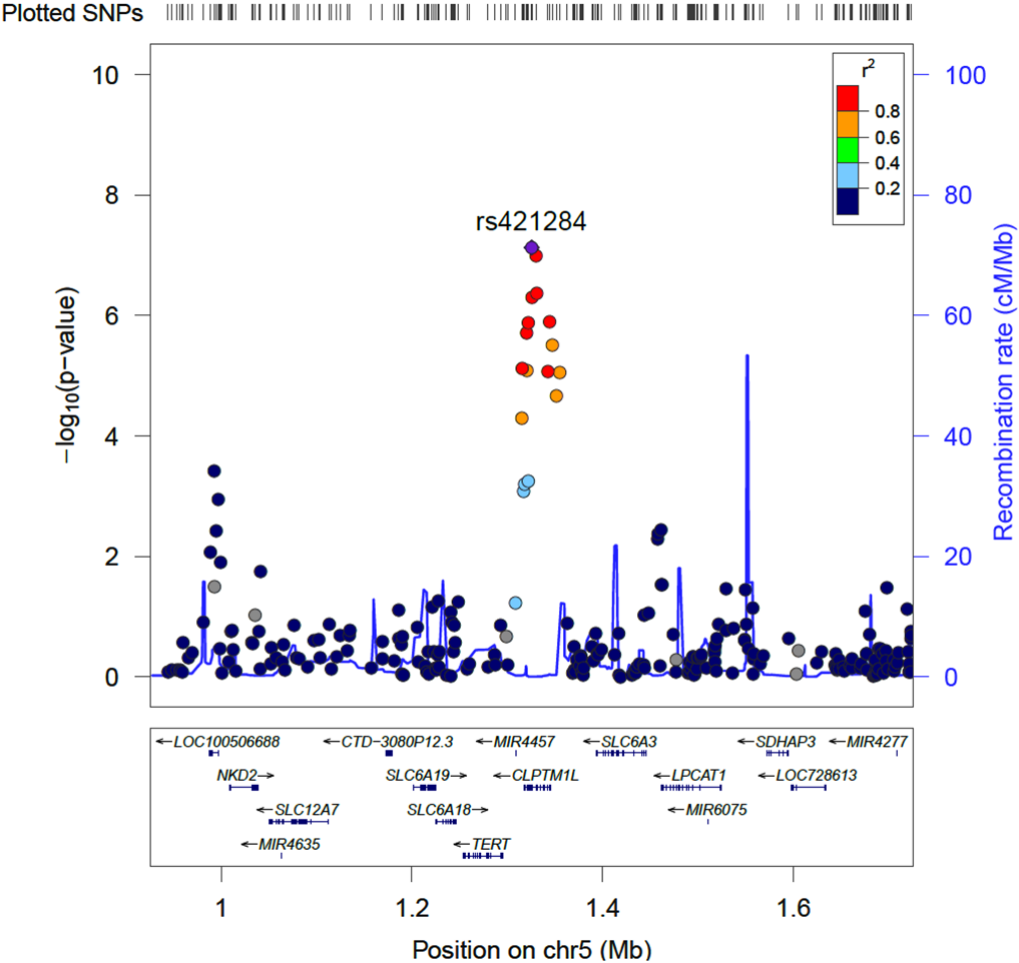
Regional linkage disequilibrium plot for 5p15.33. Genes are depicted with *blue arrows* showing transcription orientation and SNPs appear in *colored dots*. The color intensity of dots reflects the level of linkage disequilibrium with the highlighted SNP of interest (shown with a *purple diamond*). The *blue line* indicates recombination rates in the CEU population. Linkage disequilibrium (*r* ^*2*^) was calculated in the CEU population

A genome wide association study (GWAS) for choroidal UM was conducted in populations of European ancestry (see Supplementary Fig. 1 for an overview). After quality filtering removing poorly performing SNPs and DNAs, a set of 275 UM cases (UMs) and 427 French controls (CTLs) were selected using sequentially a principal component analysis (PCA) and a *K*-means (*K* = 4) clustering method. This clustering step ensured that outliers were excluded from the association study, providing a homogeneous set of UMs and CTLs (see Supplementary Fig. 2). High quality genotypes for 866,782 SNPs shared by UMs and CTLs were available for association testing. In one region on chromosome 5p15.33, 2 SNPs in high linkage disequilibrium (LD; *r* ^*2*^ > 0.9), rs421284 and rs452932, showed evidence of association with *P*-values lower than 3.3 × 10^−7^ using logistic regression (Odds ratio [OR] = 1.95, 95% CI 1.11–3.44, *P* = 7.5 × 10^−8^ and OR = 1.91, *P* = 1.1 × 10^−7^, 95% CI 1.10–3.30, respectively), while multiple surrounding SNPs showed association consistent with degradation of LD around this association peak (Figs 1 and 2, Supplementary Fig. 3 and Supplementary Table 1). A second locus showed many SNPs in LD on chromosome 15 (*OCA2/HERC2* locus) but did not reached significant threshold of 3.3 × 10^−7^. Other isolated SNPs with low *P*-value were inconsistent with surrounding SNPs in LD.

### Validation study

The validation study was performed on an independent series of 276 French UM patients and 184 CTLs of European ancestry. These samples were genotyped by TaqMan assays for the two most significant SNPs identified on the discovery series: rs421284 and rs452932 (5p15.33). These analyses confirmed the association observed in the discovery set (rs421284: OR = 1.46, 95% CI 1.11–1.91, *P* = 6 × 10^−3^ and rs452932: OR = 1.49, 95% CI 1.14–1.97, *P* = 8 × 10^−3^) for 5p15.33. Meta-analyses performed on both discovery and validation series for rs421284 and rs452932 reinforced the association observed for 5p15.33 (OR = 1.71, 95% CI 1.43–2.05, *P* = 5 × 10^−9^ and OR = 1.72, 95% CI 1.44–2.06, *P* = 2 × 10^−9^, respectively) (Table 1).

**Table 1:** Meta-analysis results for discovery and validation studies

				Discovery			Validation			Combined			
Proposed candidate	SNP	Risk allele	MAF^a^ UMs/CTLs	Nb^b^	*P*-value	OR^c^ (95% CI)	Nb^b^	*P*-value	OR^c^ (95% CI)	Nb^b^	*P*-value	OR^c^ (95% CI)	OR hom^d^ (95% CI)
*CLPTM1L*	rs421284	C	0.45/0.40	636	7 × 10^−8^	1.95 (1.11–3.44)	459	6 × 10^−3^	1.46 (1.11–1.91)	1095	5 × 10^−9^	1.71 (1.43–2.05)	3.23 (2.23–4.70)
*CLPTM1L*	rs452932	C	0.45/0.40	653	1.1 × 10^−7^	1.91 (1.10–3.30)	453	8 × 10^−3^	1.49 (1.14–1.97)	1106	2 × 10^−9^	1.72 (1.44–2.06)	3.18 (2.20–4.59)

*P-*values and odds ratio calculated using PLINK v1.07 and the R package metafor, and adjusted for sex and age

^a^ Minor Allele Frequency in uveal melanoma cases (UMs) and controls (CTLs)

^b^ Number of individuals considered for association studies

^c^ Odds ratio per-allele, Cochran–Armitage test for trend

^d^ Odds ratio of homozygotes for risk allele

### Expression Quantitative Trait Loci (eQTL) analysis

**Fig. 3:**
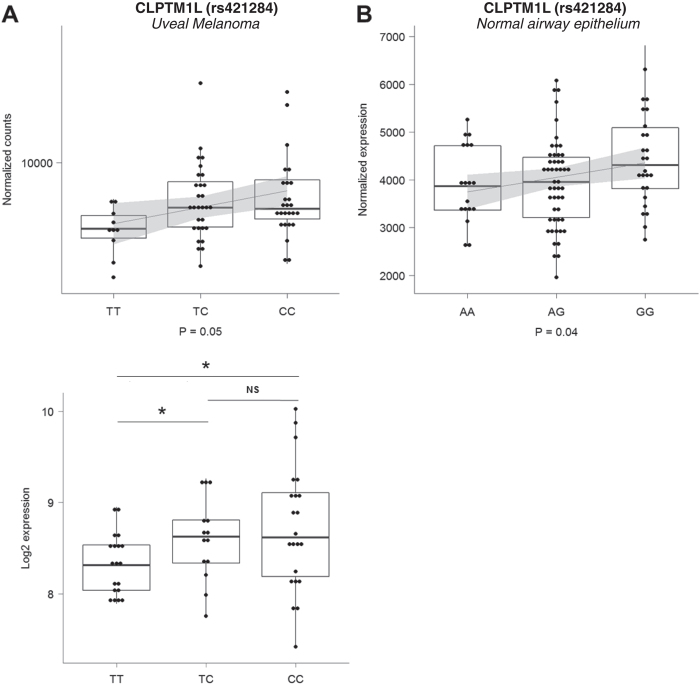
Expression of *CLPTM1L* according to SNP genotype at 5p15.33. **a** Expression QTL (eQTL) was performed for rs421284 on uveal melanoma (UM) from two series of UM patients. *Upper panel*: series described in ref. 13 *Lower panel*: series described in ref. 14 **b** Expression QTL (eQTL) was performed for rs465498 on normal airway epithelium from public dataset GSE40364. Linear regression was applied for series described in ref. 13 and GSE40364 (validated homoscedasticity), and the nonparametric Behrens–Fisher problem was applied for the series described in ref. 14 *NS* Non Significant, *P < 0.05

All 5p15.33 risk variant SNPs were found within the *TERT/CLPTM1L* locus. To evaluate the impact of SNPs on gene regulation, an eQTL analysis was performed for the 5p15.33 region using expression data from tumors of two in-house series of 73 and 55 UM patients, respectively,^14, 15^ which were genotyped for rs421284. *TERT* (Telomerase reverse transcriptase) showed very low expression in all UMs and this expression was not correlated with the genotypes. The expression of a single gene with the 500 kb region surrounding rs421284 was found correlated with the risk allele in the 2 series: *CLPTM1L* (Cleft lip and palate transmembrane protein 1-like)*,* for which a positive correlation with the risk allele was found (Fig. 3a, Supplementary Fig. 4). In addition, the influence of rs465498 on *CLPTM1L* expression was confirmed on normal airway epithelium from 95 individuals, with a higher expression associated with the risk allele of rs465498 (OR = 1.82, 95% CI 1.08–3.06, *P* = 5 × 10^−7^), in high LD with rs421284 (*r* ^*2*^ > 0.9) (Fig. 3b). Finally, we conducted an eQTL analysis on 333 cutaneous melanomas from The Cancer Genome Atlas and also showed a higher expression of *CLPTM1L* with the risk allele of rs465498 (Supplementary Fig. 5). *TERT* was weakly expressed and its expression was not correlated to rs465498 genotype.

### Haplotype analysis

To evaluate whether the variants we identified in *CLPTM1L* could explain the prevalence of UM in the different human populations, we conducted a haplotype analysis on the 5p15.33 region using HapMap populations. The risk haplotype found in European populations shows a frequency of 46% that was lower or similar to the frequency in populations of African ancestry (Luhya 55%, Maasaï 46% and Yoruba 67%) (Supplementary Table 2).

## Discussion

We conducted the first GWAS in uveal melanoma. Association analyses identified a susceptibility locus at 5p15.33 in a region showing the strongest association and including multiple SNPs in LD with rs421284 and rs452932. This locus of ~63 kb harbors two plausible candidate genes, *TERT* and *CLPTM1L*, and many SNPs in LD. The *TERT/CLPTM1L* region is frequently identified by GWAS for conferring tumor risk for many tumor types, including pancreatic, lung, melanoma, and bladder cancers (see refs 16, 17 for meta-analyses). In human cutaneous melanoma, recurrent mutations in the *TERT* promoter have previously been identified.^18^ A single UM tumor has been reported as carrying a mutation in the *TERT* promoter (chr5:1,295,226G > A) leading to elevated *TERT* expression.^19^ However, *TERT* was not significantly expressed in any of the UM tumors included in our study leading us to focus on the adjacent gene *CLPTM1L*. Some variants of *CLPTM1L* have previously been negatively or positively associated with different cancers. For example, the region containing rs451360 (intron 16 of *CLPTM1L*) is positively associated to pancreatic and testicular cancers, but negatively associated with lung cancer.^17^ Conversely, rs31489, rs401681, and rs402710 (*CLPTM1L* introns 2, 13, and 16, respectively) have been associated by multiple GWAS with several cancer types such as cutaneous melanoma, bladder, pancreatic, and lung carcinomas.^20–24^ In particular, rs401681 shows an inverse association with cutaneous melanoma and may change its risk via the variation of nevus counts.^16, 24^ This SNP is part of the *CLPTM1L* peak detected in our GWAS (Supplementary Table 1) and is in high LD with rs421284 (*r* ^*2*^ = 0.93).

Our eQTL analyses revealed a positive correlation between risk allele of rs421284 and *CLPTM1L* expression in UM tumors. In normal tissue, the risk allele of rs465498, another SNP of the region, was also found to be positively correlated with *CLPTM1L* expression. In accordance with our results, James and colleagues have previously reported a correlation between *CLPTM1L* expression and rs31489 alleles in normal lung tissue.^25^ rs31489 was part of the peak at 5p15.33 and is in high LD (*r* ^*2*^ = 0.8) with rs421284. In addition, we showed a positive correlation between the rs465498 risk allele and *CLPTM1L* expression in cutaneous melanoma. Interestingly, the SNPs we discovered with the highest OR at 5p15.33 are located close to or within a region highly marked for H3K27ac (ENCODE) and associated with DNase I hypersensitivity clusters in *CLPTM1L* intron 8, both of which are indicative of an active enhancer region (Supplementary Fig. 6). This suggests that one variant included in or closely linked to the identified SNPs could positively modify this regulatory region. It should be highlighted that if *CLPTM1L* contributes to UM risk in European population, it does not explain the striking difference with populations of African ancestry where the prevalence of risk alleles were found at similar or higher frequencies as compared with populations of European origin.

The function of *CLPTM1L* is not yet fully understood. *CLPTM1L* is expressed at higher levels in cisplatin ovarian resistant cell lines and doxorubicin resistant breast tumors.^26, 27^ *CLPTM1L* expression was shown to confer resistance to chemotherapy and to anoikis in experimental models, potentially by regulating the BCL-xL pathway.^28, 29^ *CLPTM1L* was also shown to contribute to RAS-dependent transformation and tumorigenesis by its interaction with phosphoinositide 3-kinase (PIK3CA).^25^ UM malignant transformation is dependent of activating mutations of *GNAQ* or *GNA11,* whereas *RAS* family members are rarely found mutated.^1, 2^ Whether *CLPTM1L* may also be involved in *GNAQ/GNA11*-dependent transformation and how this could be connected to the RAS and PI3K pathways remains to be elucidated.

Despite the fact that both are derived from neural crest, there are major differences between uveal and cutaneous melanoma in terms of genetics. High penetrance susceptibility genes for cutaneous melanoma such as *CDKN2A* and *CDK4* are not predisposing for uveal melanoma.^30, 31^ The reverse is less clear: *BAP1,* the only known high penetrance susceptibility gene for uveal melanoma, also predisposes to cutaneous melanoma, mostly of atypical/low malignity types.^32–34^ Cutaneous melanoma GWAS identified loci containing genes that are implicated in one of the two well-established heritable risk phenotypes for melanoma, nevus count (*CDKN2A*/*MTAP*, *PLA2G6*, and *TERT*) and pigmentation (*SLC45A2*, *TYR*, *MC1R*, *ASIP*).^35, 36^ In our discovery series, most of these loci displayed low ORs (0.74 < OR < 1.20) and were far from the significance threshold (Supplementary Table 3). Nevertheless, rs4911442 in the *NCAO6/ASIP* region and associated with cutaneous melanoma,^37^ exhibited a high OR (OR = 1.77, 95% CI 0.90–3.50, *P* = 5.6 × 10^−3^) in our study, but did not achieve the significance threshold. While this study was completed, the association between *HERC2/OCA2* and *IRF4* pigmentation genes in cutaneous melanoma was also found for UM risk.^11^ Authors described rs12913832 at the *HERC2/OCA2* locus as the most significantly associated with UM risk. Interestingly, three SNPs at the *HERC2/OCA2* locus, rs12913832, rs11074306, and rs3930739 displayed a clear peak in the Manhattan plot in our study, although not reaching our empirical significance threshold (Supplementary Table 1). A combined risk analyses was performed between *OCA2* alleles and rs421284. No interaction between these risk loci was evidenced. However, the confidence intervals are too wide for any conclusion. (Supplementary Table 4). Ferguson and colleagues also showed that rs12203592 at the *IRF4* locus was associated with UM risk. This SNP was called in our GWAS with similar odds ratio (OR 1.88, 95% CI 1.03–3.44, *P* = 2.5 × 10^−5^), although not reaching statistical significance (Supplementary Table 1). To be noticed, Ferguson and colleagues also evaluated rs401681 located in the *CLPTM1L* locus, which did not reach significance in their study. However, the OR confidence intervals of both UM studies overlap for rs401681, and Ferguson’s study was not stratified for ancestry origin and for choroidal UM, which may explain their lower OR. Thus, our analysis supports the report by Ferguson and colleagues, indicating that although cutaneous and uveal melanoma have striking differences in terms of oncogenic events, they share some genetic predisposition factors. It is intriguing that pigmentation genes are associated with UM risk, while we previously demonstrated the absence of a role of UV-dependent mutagenesis in this disease.^4^ One possibility is that these pigmentation variants reflect a population bias in UM patients, which escaped the PCA stratification. We thus compared the UM and CTL discovery series with different European sub-populations from the Human Genome Diversity Panel and excluded such bias (*P* = 0.62; Supplementary Fig. 7). By which mechanisms pigmentation gene polymorphisms contribute to UM epidemiology remain to be unraveled. Roles of melanin beyond UV protection are emerging, including scavenging reactive oxygen or possibly modulating the inflammatory response and should be further investigated in the future.^38^

In conclusion, this first GWAS in uveal melanoma suggests a susceptibility allele for UM in or around the *CLPTM1L* locus. Further work will be necessary to determine the significance of these findings on biological mechanisms underlying UM oncogenesis.

## Methods

### Study populations

This study was approved by the Ethical committee and Internal Review Board at the Institut Curie, and blood (germline) samples were obtained from uveal melanomas patients (UMs)—all choroidal cases but two: one iris case and one ciliary body case-, who consented to participate to the study. Patient constitutional DNA samples (277 and 276 UM patients for discovery and validation series, respectively) of adequate quality were obtained. Main clinical characteristics of UM patients from the discovery series is provided (Supplementary Table 5). A brief description of the Genomic DNA was isolated from blood using DNeasy Blood & Tissue Kit (Qiagen) according to the manufacturer’s instructions. Genome-wide genotyping was performed on the discovery series using the Illumina HumanOmni5 platform at the *Centre National de Génotypage* (Evry, France). Genotypes were called using default parameters in GenomeStudio (Illumina).

Controls were selected from among a parallel GWAS on renal cell carcinoma (US NCI U01CA155309; G. Scelo). Non-cancer participating individuals from France that had been genome-wide genotyped using the Illumina HumanOmni5 platform contributed to the discovery phase. An additional 184 participants were selected for the validation phase. Our study was designed such that for a minor allele frequency >0.1 and a *P*-value ≤10^−6^, our power to detect a per-allele relative risk of at least 2 is 80%.^39^

### Quality control in the discovery series

The discovery series (UM and CTL) was filtered using the same criteria in an automated pipeline, briefly described here. All SNPs were filtered, mapped and synchronized with respect to strand. dbSNP146 on human genome build 37.1 (GRCh37) was used as the reference map. Only SNPs mapping to a single unique location on GRCh37 with only two alleles were included in analyses. Using PLINK v1.07,^40^ SNPs or subjects yielding a genotype completion of <95% were filtered out from analyses (one CTL participant). SNPs were then filtered for departure from Hardy–Weinberg equilibrium at *P* < 0.001 in the control series, and SNPs with a minor allele frequency < 0.05 were filtered out. Three pairs of subjects presented cryptic relatedness (identity by state >30%): one sample within each pair was then randomly selected and excluded for the analysis. Details regarding the number of SNPs and DNA samples filtered at each step of data preparation are provided in Supplementary Fig. 1. After all filtering, 702 subjects and 866,782 SNPs were kept in the discovery data set.

### Sample selection to limit population stratification

PCA was used to select UMs and CTLs for association testing. To select the most representative subjects from the European population, discovery data were merged with the HapMap data using PLINK v1.07 (1744 subjects and 427,139 SNPs) and submitted to PCA analysis using R version 3.2.4. *K*-mean analysis enabled the selection of 259 UMs and 401 CTLs for subsequent analyses. To ensure the origin of those 660 individuals, they were also merged with European populations from the Human Genome Diversity Panel (Dataset 2 from Stanford University,^41^) using PLINK v1.07.

### Statistical analyses

Unconditional logistic regressions were carried out in PLINK v1.07 using the Cochran–Armitage test for trend. Each SNP was coded as a quantitative variable, with values zero, one and two corresponding to the number of variant alleles carried. Age and sex and the first 2 principal components were included in the models.

Inflation factors based on quantile–quantile (Q–Q) plots were calculated using the “qqman” package in R. The Q–Q plots comparing the distribution of *P*-values observed to those expected show little evidence of any residual population stratification in either analysis (Supplementary Fig. 3; UMs vs. CTLs inflation factor 1.02). The regional linkage disequilibrium plot was made with the LocusZoom software.^42^ Significance was declared at *P* < 3.3 × 10^−7^. Results for SNPs with a *P*-value < 1 × 10^−5^ (suggestive line) are shown in Supplementary Table 1.

Difference in distribution between UMs and CTLs on PCA with European individuals from the Human Genome Diversity Panel was evaluated using a multivariate Kolmogorov–Smirnov test. Significance was declared at *P* < 0.05.

### Haplotype analyses

The most likely haplotypes for 5p15.33 of each individual of the discovery series was estimated using SHAPEIT v2^43^ with default parameters and the 1000 genomes data as reference panel. The haplotypes were then narrowed to the 8 SNPs in *CLPTM1L* that are common between our series and the HapMap data. The haplotypes relative frequencies in the discovery series were then compared to the haplotype relative frequencies of the HapMap populations (HapMap3 release #2, phased data).

### Validation study

For the validation, SNPs were genotyped using TaqMan allele-specific probes and PCR primers (Supplementary Table 6). PCRs were carried out according to the manufacturer’s instructions on 15 ng of genomic DNA using the following cycling conditions: initial denaturation at 95 °C for 10 min; 40 cycles of 95 °C for 15 s, 60 °C for 1 min. Endpoint analyses were carried out using the Applied Biosystems 7500HT Fast Real-Time PCR System and collected using Applied Biosystems TaqMan Genotyper Software Version 1.3. Unconditional logistic regressions were carried out in R using the Cochran–Armitage test for trend. Each SNP was coded as a quantitative variable, with values zero, one and two corresponding to the number of variant alleles carried. Age and sex were included in the models.

### Meta-analysis

A meta-analysis was performed on the discovery and validation series using R package metafor.^44^ The combined OR together with their 95% confidence intervals were assessed with the random-effects method. The between-study heterogeneity was estimated by the χ^2^-based Q test (significance level, *P* < 0.10). Results were adjusted for age and sex.

### Expression analyses

A total of 128 UM tumor samples from UM patients treated at Institut Curie were used to perform an eQTL analyses. For the 73 UM tumor samples,^14^ RNA-seq data were simultaneously normalized using the DESeq2 package^45^ version 1.10.0 in R version 3.2.2. For the 55 UM tumor samples,^15^ microarray data were normalized by the log_2_ of gene expression. To define an expression threshold, all local minima of the median distribution were searched for each gene. The threshold was set at 67.3 and 4.9 for RNA-seq and microarray normalized data, respectively. As validation, eQTL was performed on normal airway epithelium (95 tissues) from public data from Gene Expression Omnibus, GSE40364. Finally, eQTL was performed on cutaneous melanoma from The Cancer Genome Atlas (http://cancergenome.nih.gov/). Individuals with copy number alteration at 5p15.33 were removed from analyses. Correlation between genotype and expression was examined with linear regressions using the lm function in R v3.2.4. To apply the lm function, homoscedasticity was tested using a Breusch–Pagan test using the lmtest package in R. If not met, the nonparametric Behrens–Fisher problem was used using the R package nparcomp to detect significant change in gene expression between each genotype.^46^ Significance was declared at *P* < 0.05.

## Accession codes

The genotype data of cases are deposited on the European Genome-Phenome Archive (EGA) under Accession number EGAS00001002334. The genotype data of controls are deposited on the database for Genotypes and Phenotypes (dbGaP) under accession number phs001271.v1.p1.

## Electronic supplementary material

Supplementary Information
