# Supplementary material for: A GWAS in uveal melanoma identifies risk polymorphisms in the CLPTM1L locus
Source: NPJ Genom Med. 2017 Mar 10;2:5. doi: 10.1038/s41525-017-0008-5 (PMC5542017; doi:10.1038/s41525-017-0008-5)
Supplement: Supplementary file 1 — Supplementary Information [file 41525_2017_8_MOESM1_ESM.docx]

**Supplementary Fig 1.** Flow chart of the GWAS analyses.


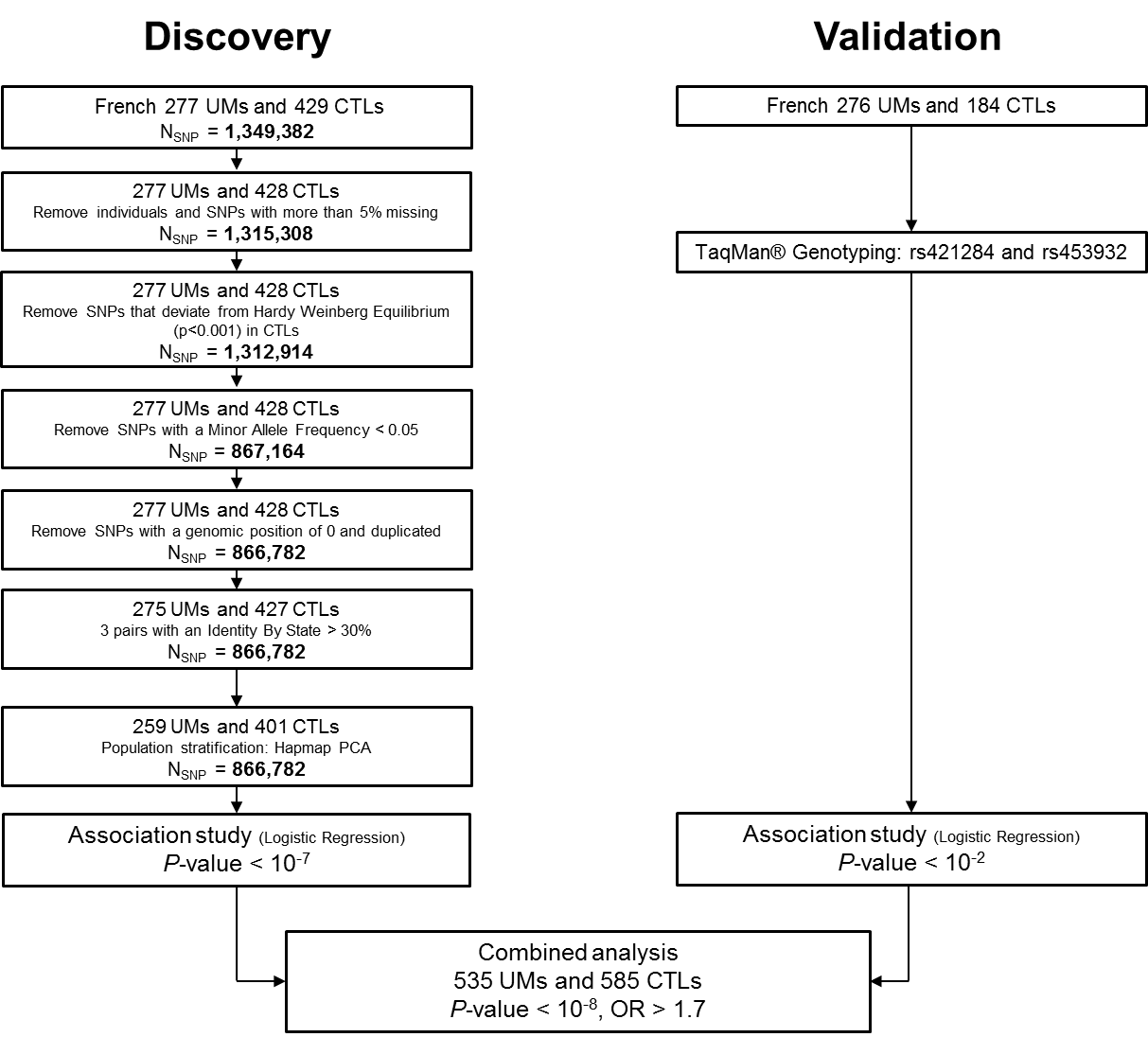


UMs: Uveal melanoma patients, CTLs: Controls

**Supplementary Fig 2.** Clustering to select population for subsequent association analysis.


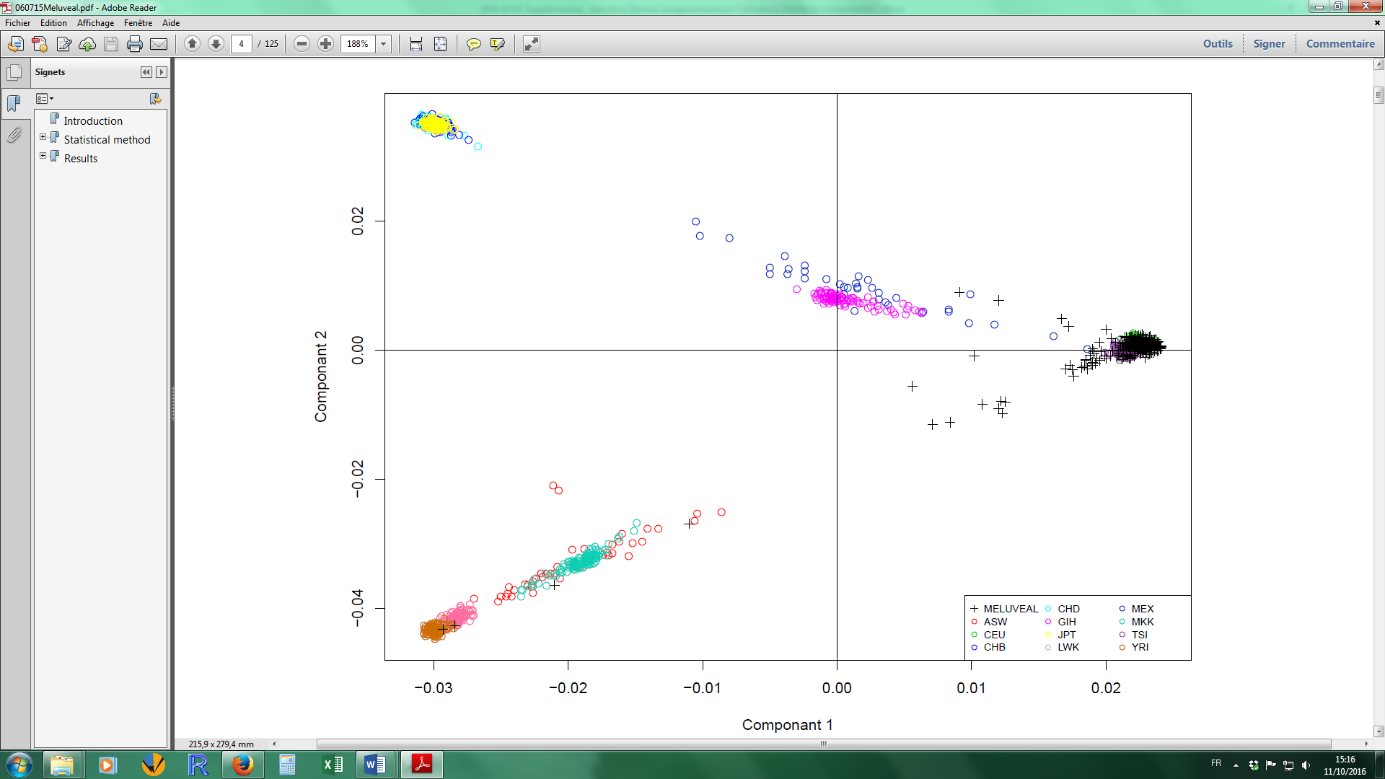
**A**


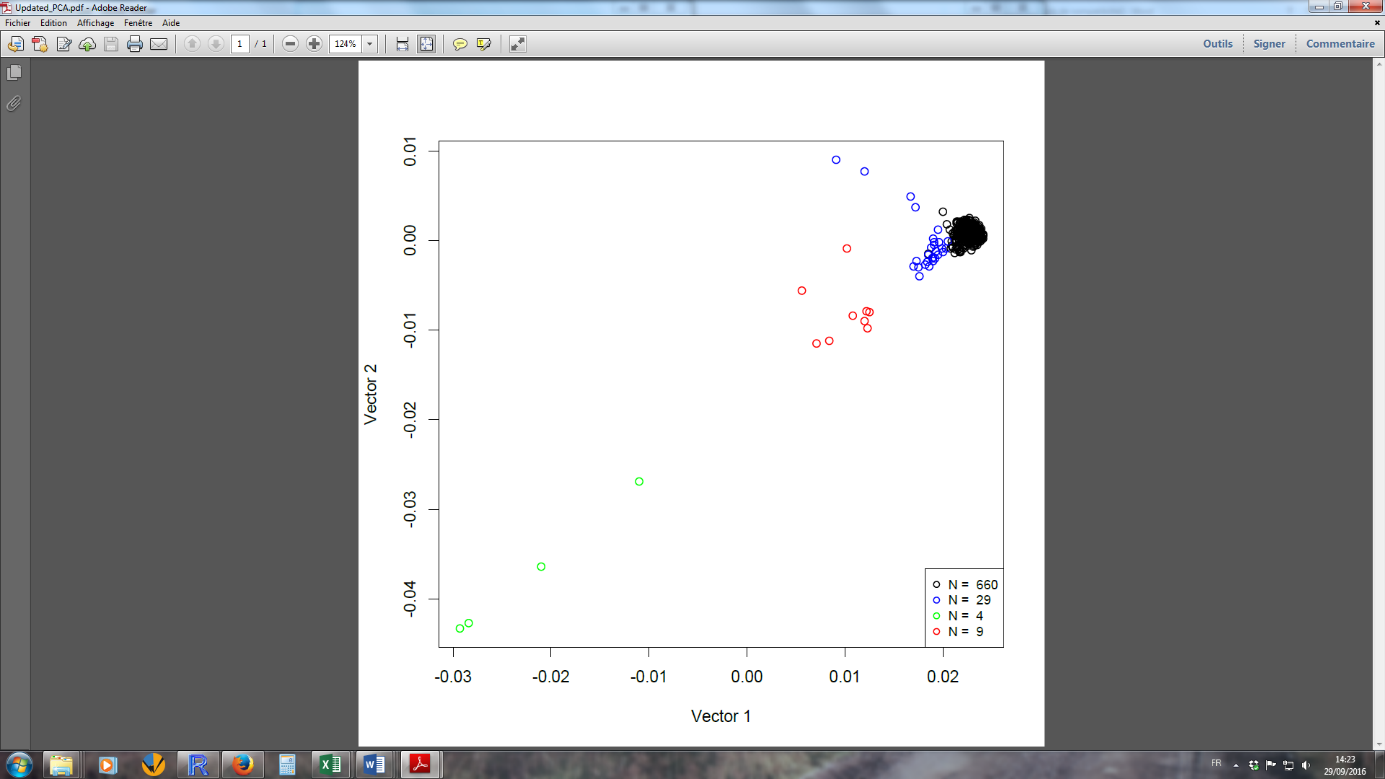


**B**

1. Clustering projected on the first two PCA axes for the discovery series vs HapMap populations.
2. The discovery series projected on the first two axes of the PCA. Individuals in black were kept for the association analysis.

**Supplementary Fig 3.** Q-Q plot for the GWAS.

The red line represents the theoretical distribution of quantiles of the statistical association test while the black points show the real distribution, and the grey area represents the 95% confidence interval around the distribution of *P*-values.


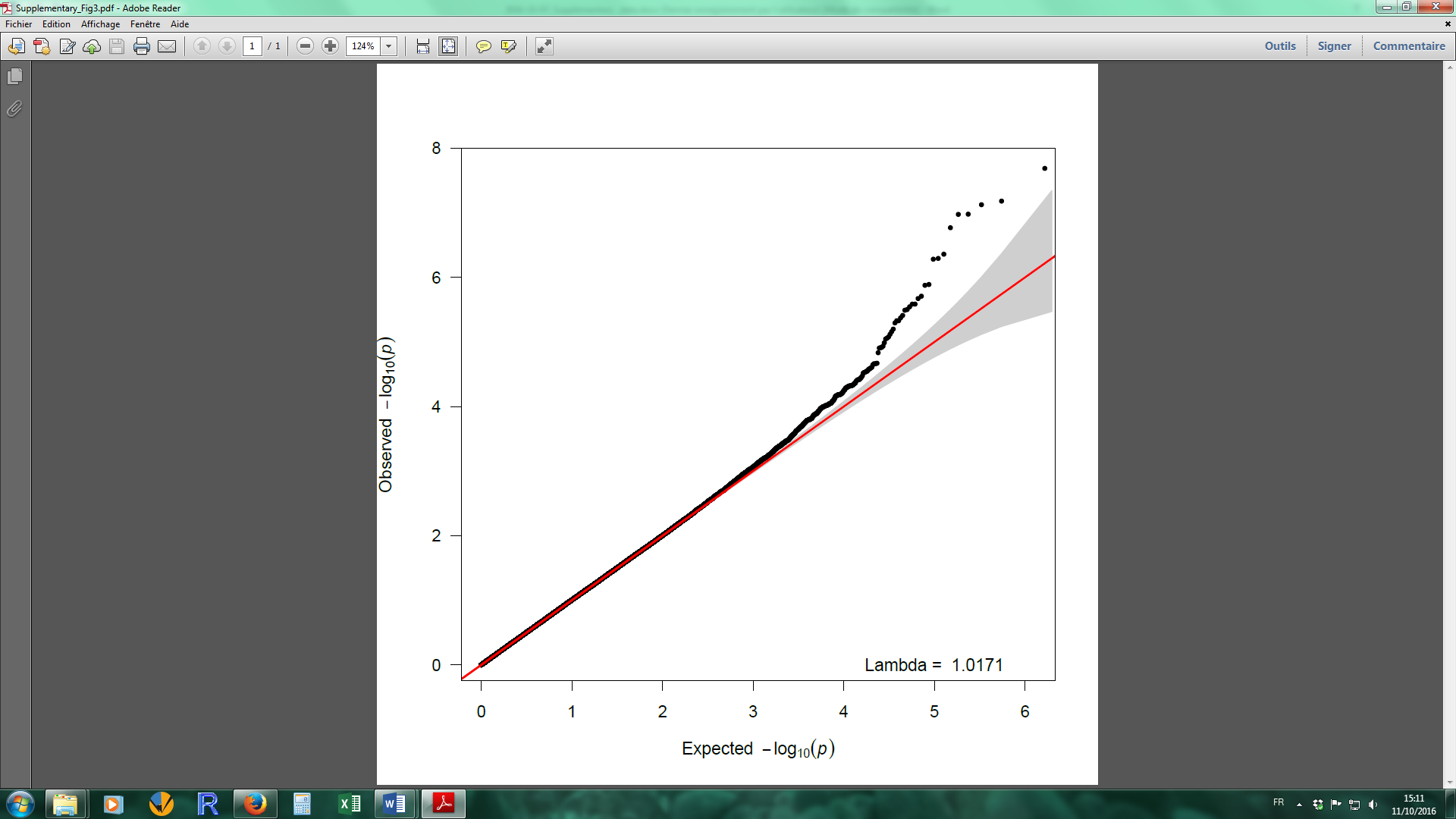


**Supplementary Fig 4.** eQTL analysis on 5p15.33 region (500 kb around *CLPTM1L*) according to rs421284 genotypes.

Correlation between genotypes and gene expression was evaluated using a linear regression. ns: not significant.

**
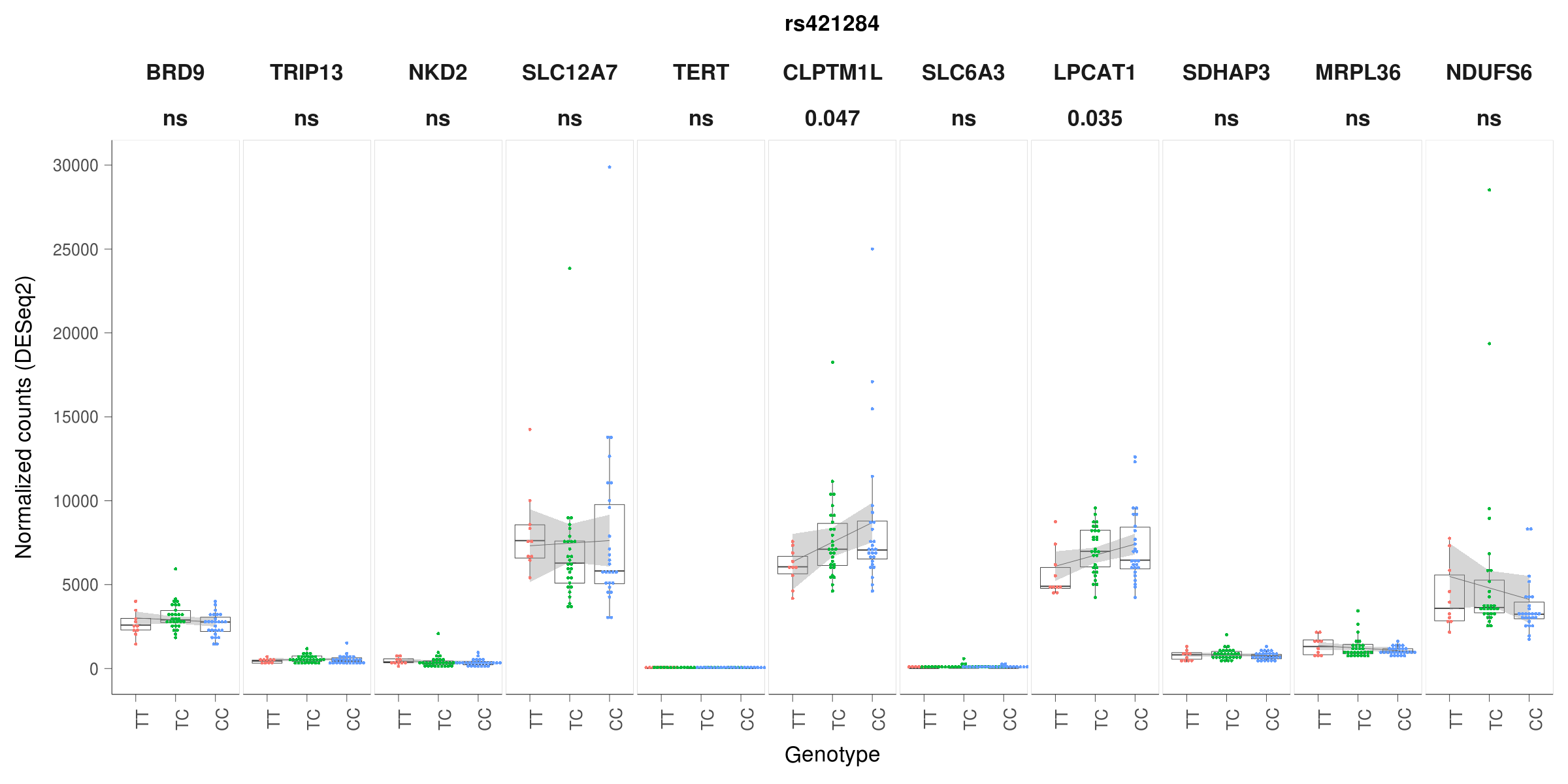
**

**Supplementary Fig 5.** Expression of *CLPTM1L* and *TERT* according to rs465498 genotypes in cutaneous melanoma tumors.

Data were extracted from The Cancer Genome Atlas for cutaneous melanoma (<http://cancergenome.nih.gov/>). Patients with copy number alterations were removed from the analysis and eQTL was evaluated using a linear regression.


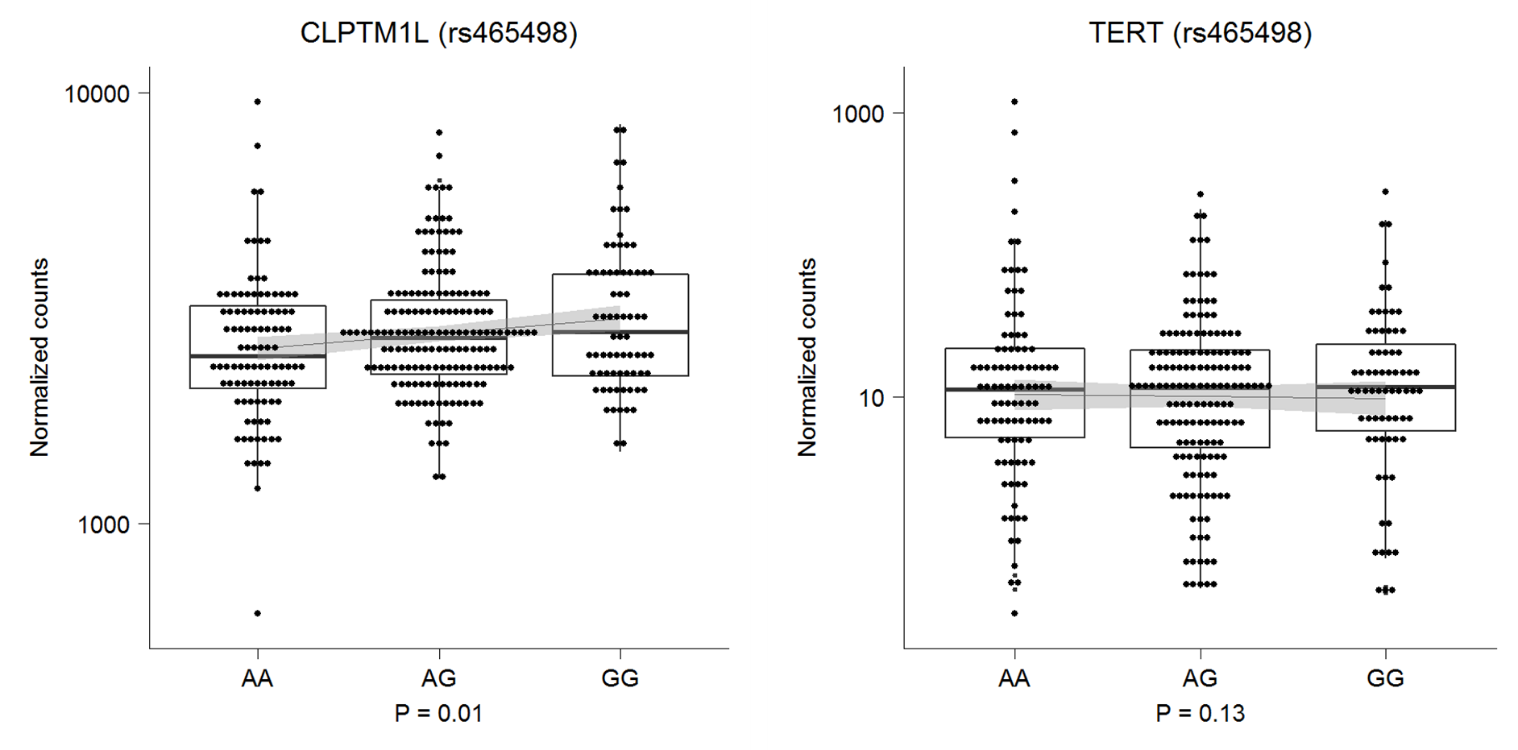


**Supplementary Fig 6.** ENCODE marks and DNase I clusters susceptibility at 5p15.33.

The figure was downloaded from UCSC genome browser on Hg19 (<https://genome.ucsc.edu/> as 10/04/2016). SNPs discovered in this GWAS are indicated, SNPs with odds ratio >1.8 in red and the others in orange.

**
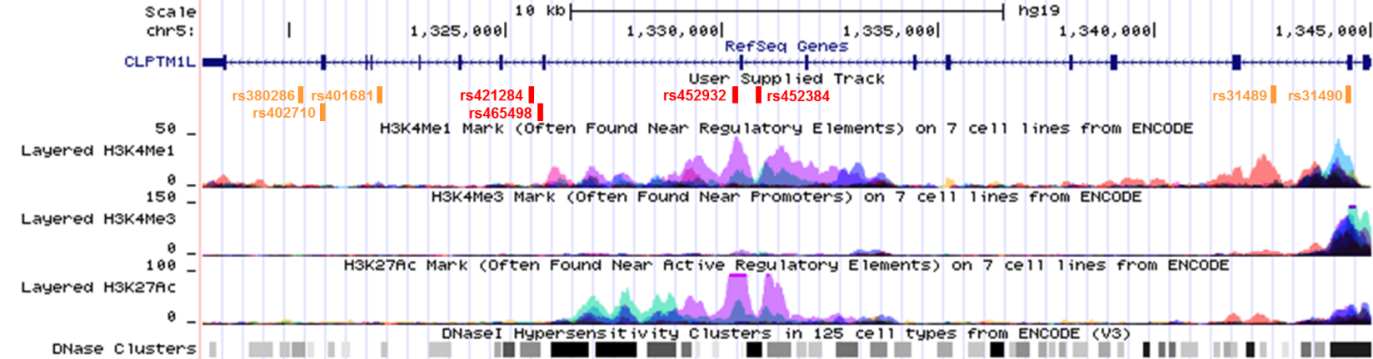
**

**Supplementary Fig 7.** Population stratification by Principal Component analysis.

Projection of the discovery series on the Principal Component analysis (PCA) of the reference European populations from Human Genome Diversity Panel. Data were merged and PCA coordinates were calculated using PLINK v1.07. The heterogeneity between UMs and CTLs was tested using a multivariate Kolmogorov-Smirnov test, showing no difference between both series (*P* = 0.62).


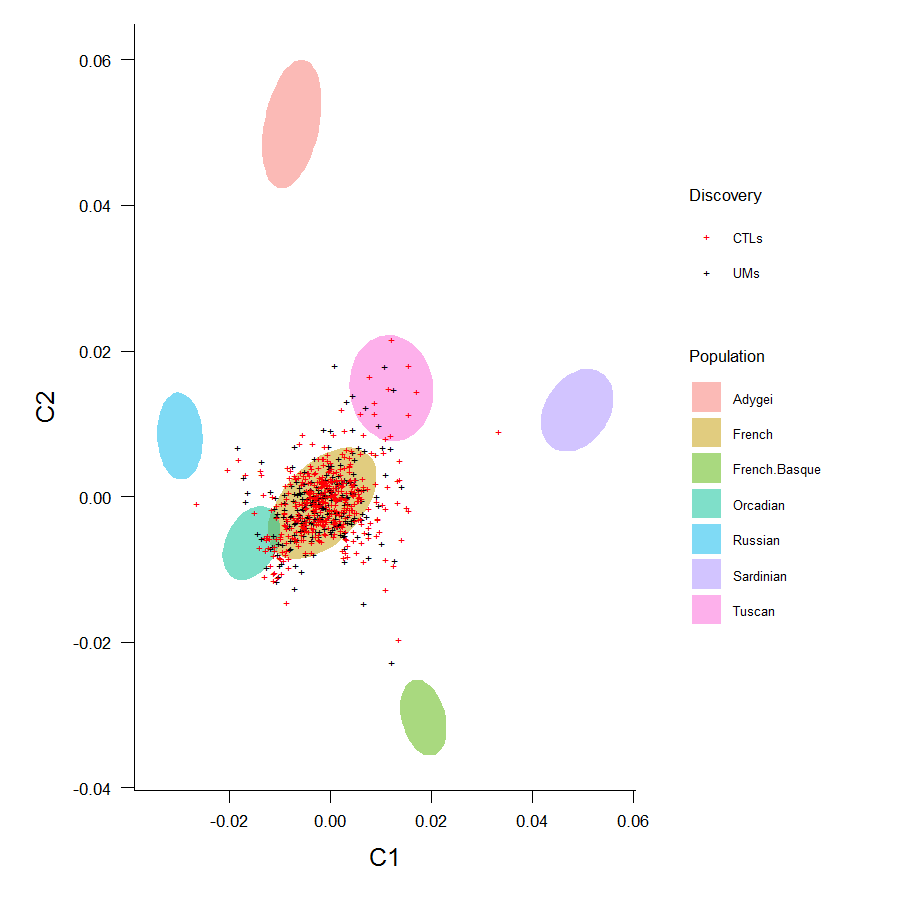


**Supplementary Table 1.** List of SNPs with *P*-values < 1 x 10^-5^ for association test with uveal melanoma risk.

Significance was measured using unconditional logistic regressions and the Cochran-Armitage test for trend.

| **SNP** | **CHR** | **Position** | **Risk Allele** | **RAF (UMs/CTLs)** | **NB** | **OR** | **95% CI** | ***P*-value** |
| --- | --- | --- | --- | --- | --- | --- | --- | --- |
| rs77612181 | 4 | 79593944 | G | 0.12/0.007 | 654 | 11.49 | 1.52-86.68 | 2.05 x 10^-8^ |
| rs200446504 | 10 | 55943362 | A | 0.008/0.12 | 630 | 0.06 | 0.005-0.64 | 6.58 x 10^-8^ |
| **rs421284** | 5 | **1325590** | **C** | **0.45/0.40** | **636** | **1.95** | **1.11-3.44** | **7.48 x 10^-8^** |
| **rs452932** | 5 | **1330253** | **C** | **0.45/0.40** | **653** | **1.91** | **1.10-3.30** | **1.05 x 10^-7^** |
| rs1758138 | 6 | 108705377 | G | 0.42/0.41 | 628 | 1.88 | 1.10-3.20 | 1.06 x 10^-7^ |
| rs1906012 | 2 | 180675669 | C | 0.36/0.23 | 639 | 2.03 | 1.11-3.72 | 1.70 x 10^-7^ |
| **rs452384** | 5 | **1330840** | **C** | **0.45/0.40** | **660** | **1.83** | **1.08-3.09** | **4.36 x 10^-7^** |
| **rs465498** | 5 | **1325803** | **G** | **0.45/0.41** | **660** | **1.82** | **1.08-3.06** | **5.08 x 10^-7^** |
| rs11074306 | 15 | 28044542 | A | 0.37/0.51 | 660 | 0.54 | 0.31-0.92 | 5.22 x 10^-7^ |
| **rs31490** | 5 | **1344458** | **A** | **0.45/0.40** | **659** | **1.78** | **1.06-2.97** | **1.29 x 10^-6^** |
| **rs401681** | 5 | **1322087** | **T** | **0.45/0.41** | **660** | **1.78** | **1.06-2.96** | **1.32 x 10^-6^** |
| **rs380286** | 5 | **1320247** | **A** | **0.45/0.41** | **660** | **1.76** | **1.06-2.92** | **1.95 x 10^-6^** |
| rs3930739 | 15 | 28040342 | T | 0.46/0.34 | 660 | 1.79 | 1.06-3.03 | 2.12 x 10^-6^ |
| rs4238494 | 15 | 28041939 | C | 0.46/0.34 | 659 | 1.79 | 1.06-3.01 | 2.59 x 10^-6^ |
| rs4778189 | 15 | 28072228 | A | 0.48/0.35 | 660 | 1.78 | 1.06-3.00 | 2.59 x 10^-6^ |
| rs6497235 | 15 | 28049304 | A | 0.44/0.32 | 660 | 1.78 | 1.05-3.01 | 2.85 x 10^-6^ |
| **rs27069** | 5 | **1347128** | **T** | **0.47/0.40** | **643** | **1.75** | **1.05-2.92** | **3.14 x 10^-6^** |
| rs4778192 | 15 | 28080061 | C | 0.48/0.35 | 660 | 1.77 | 1.05-2.96 | 3.22 x 10^-6^ |
| rs1498509 | 15 | 28030000 | A | 0.49/0.37 | 660 | 1.77 | 1.05-2.96 | 3.88 x 10^-6^ |
| rs9567130 | 13 | 43762719 | A | 0.22/0.13 | 660 | 2.05 | 1.06-3.96 | 4.22 x 10^-6^ |
| rs201671914 | 15 | 28068549 | A | 0.47/0.35 | 660 | 1.75 | 1.05-2.91 | 4.68 x 10^-6^ |
| rs2311469 | 15 | 28069068 | T | 0.47/0.35 | 660 | 1.75 | 1.05-2.91 | 4.68 x 10^-6^ |
| rs884197 | 15 | 28027100 | A | 0.49/0.37 | 658 | 1.76 | 1.05-2.94 | 5.06 x 10^-6^ |
| rs4709827 | 6 | 164599359 | A | 0.39/0.28 | 660 | 1.79 | 1.05-3.06 | 6.31 x 10^-6^ |
| rs201104404 | 11 | 65539305 | G | 0.51/0.39 | 631 | 1.79 | 1.04-3.06 | 6.92 x 10^-6^ |
| **rs4975616** | 5 | **1315660** | **G** | **0.48/0.39** | **660** | **1.70** | **1.04-2.78** | **7.60 x 10^-6^** |
| **rs402710** | 5 | **1320722** | **T** | **0.42/0.30** | **656** | **1.73** | **1.04-2.86** | **8.34 x 10^-6^** |
| **rs31489** | 5 | **1342714** | **A** | **0.49/0.38** | **660** | **1.69** | **1.04-2.76** | **8.71 x 10^-6^** |
| **rs37006** | 5 | **1355058** | **T** | **0.46/0.41** | **660** | **1.69** | **1.04-2.74** | **9.00 x 10^-6^** |

SNP: single nucleotide polymorphism; CHR: chromosome; Position: chromosome position in base pair; RAF: Risk Allele Frequency for uveal melanoma cases (UMs) and controls (CTLs); NB: Number of individual in the association analysis; OR: Odds Ratio; 95% CI: Confidence Interval at 95%. SNPs located in the 5p15.33 locus are in bold.

**Supplementary Table 2.** Haplotype analysis for 5p15.33 region.

| **Haplotype** | **UMs** | **CTLs** | **UMs+CTLs** | **ASW** | **CEU** | **CHD** | **GIH** | **JPT+CHB** | **LWK** | **MKK** | **TSI** | **YRI** |
| --- | --- | --- | --- | --- | --- | --- | --- | --- | --- | --- | --- | --- |
| CT | 0 | 0 | 0 | 0 | 0 | 0 | 0 | 0.01 | 0 | 0 | 0.01 | 0 |
| TT | 0.44 | 0.59 | 0.53 | 0.38 | 0.62 | 0.85 | 0.79 | 0.83 | 0.44 | 0.53 | 0.55 | 0.33 |
| TC | 0 | 0 | 0 | 0 | 0 | 0 | 0 | 0 | 0 | 0 | 0 | 0 |
| **CC** | **0.55** | **0.4** | **0.46** | **0.54** | **0.38** | **0.15** | **0.21** | **0.15** | **0.55** | **0.46** | **0.43** | **0.67** |
| *Total** | *518* | *802* | *1320* | *26* | *34* | *170* | *176* | *340* | *180* | *174* | *176* | *18* |
|  |  |  |  |  |  |  |  |  |  |  |  |  |
| TGCC | 0 | 0 | 0 | 0 | 0 | 0 | 0 | 0 | 0 | 0 | 0 | 0 |
| TATT | 0.44 | 0.59 | 0.53 | 0.38 | 0.62 | 0.85 | 0.79 | 0.83 | 0.44 | 0.53 | 0.55 | 0.33 |
| CGTT | 0 | 0 | 0 | 0 | 0 | 0 | 0 | 0.01 | 0 | 0 | 0 | 0 |
| CATT | 0 | 0 | 0 | 0 | 0 | 0 | 0 | 0 | 0 | 0 | 0.01 | 0 |
| CACC | 0 | 0 | 0 | 0.08 | 0 | 0 | 0 | 0 | 0 | 0 | 0 | 0 |
| TACC | 0 | 0 | 0 | 0 | 0 | 0 | 0 | 0 | 0 | 0 | 0.01 | 0 |
| CGCT | 0 | 0 | 0 | 0 | 0 | 0 | 0.01 | 0 | 0 | 0 | 0 | 0 |
| ***CGCC*** | ***0.55*** | ***0.4*** | ***0.46*** | ***0.54*** | ***0.38*** | ***0.15*** | ***0.2*** | ***0.15*** | ***0.55*** | ***0.46*** | ***0.43*** | ***0.67*** |
| Total* | *518* | *802* | *1320* | 26 | 34 | 170 | 176 | 340 | 180 | 174 | 176 | 18 |
|  |  |  |  |  |  |  |  |  |  |  |  |  |
| TTCACCCG | 0 | 0 | 0 | 0.04 | 0 | 0 | 0 | 0 | 0 | 0 | 0 | 0 |
| CTCGCCCG | 0 | 0 | 0 | 0.11 | 0 | 0 | 0 | 0 | 0.07 | 0.05 | 0 | 0 |
| CTCGCCCA | 0.03 | 0.02 | 0.03 | 0.04 | 0 | 0 | 0.04 | 0 | 0.08 | 0.03 | 0.03 | 0.05 |
| CCTACCAA | 0 | 0 | 0 | 0 | 0 | 0 | 0 | 0 | 0 | 0 | 0.01 | 0 |
| CTTATTCG | 0 | 0 | 0 | 0.04 | 0 | 0.12 | 0 | 0.16 | 0 | 0 | 0 | 0 |
| CTTGCCAA | 0 | 0 | 0 | 0 | 0 | 0 | 0 | 0 | 0 | 0 | 0 | 0 |
| CCTATTAA | 0 | 0 | 0 | 0 | 0 | 0 | 0 | 0 | 0 | 0 | 0 | 0 |
| TTCGCCAA | 0.21 | 0.14 | 0.17 | 0.19 | 0.15 | 0.04 | 0.07 | 0.05 | 0.12 | 0.15 | 0.15 | 0.17 |
| CTCGCCAG | 0 | 0 | 0 | 0 | 0 | 0 | 0 | 0 | 0.01 | 0 | 0 | 0.05 |
| TTCGCCAG | 0 | 0 | 0 | 0 | 0 | 0 | 0 | 0 | 0 | 0 | 0 | 0 |
| TTCACCAA | 0 | 0 | 0 | 0 | 0 | 0 | 0 | 0 | 0 | 0 | 0 | 0 |
| TCCGCCAA | 0 | 0 | 0 | 0 | 0 | 0 | 0 | 0 | 0 | 0 | 0 | 0 |
| CCTATTCA | 0 | 0 | 0 | 0 | 0 | 0.01 | 0 | 0 | 0 | 0 | 0 | 0 |
| CTCACCCG | 0 | 0 | 0 | 0.04 | 0 | 0 | 0 | 0 | 0 | 0 | 0 | 0 |
| CTCACCAA | 0 | 0 | 0 | 0 | 0 | 0 | 0 | 0 | 0 | 0 | 0 | 0 |
| CCTATTAG | 0 | 0 | 0 | 0.04 | 0 | 0 | 0 | 0 | 0 | 0 | 0 | 0 |
| TTCGCTAA | 0 | 0 | 0 | 0 | 0 | 0 | 0.01 | 0 | 0 | 0 | 0 | 0 |
| CCCATTCG | 0 | 0 | 0 | 0 | 0 | 0 | 0 | 0 | 0 | 0 | 0.01 | 0 |
| TTTGCCAA | 0 | 0 | 0 | 0 | 0 | 0 | 0 | 0 | 0 | 0.01 | 0 | 0 |
| CCTATTCG | 0.44 | 0.58 | 0.53 | 0.31 | 0.62 | 0.72 | 0.79 | 0.67 | 0.44 | 0.53 | 0.55 | 0.33 |
| CCCGCCAA | 0.01 | 0 | 0 | 0 | 0 | 0 | 0 | 0 | 0 | 0 | 0.02 | 0 |
| CCCGTTCG | 0 | 0 | 0 | 0 | 0 | 0 | 0 | 0.01 | 0 | 0 | 0 | 0 |
| TTCGCCCG | 0.01 | 0 | 0 | 0 | 0.03 | 0 | 0 | 0 | 0 | 0 | 0 | 0 |
| ***CTCGCCAA*** | ***0.29*** | ***0.23*** | ***0.25*** | ***0.19*** | ***0.2*** | ***0.11*** | ***0.1*** | ***0.1*** | ***0.28*** | ***0.23*** | ***0.23*** | ***0.39*** |
| Total* | *518* | *802* | *1320* | 26 | 34 | 170 | 176 | 340 | 180 | 174 | 176 | 18 |

*Number of haplotype. Haplotype analyses for rs421284 and rs452932, for rs421284, rs465498, rs452932 and rs452384, and for rs402710, rs401681, rs421284, rs465498, rs452932, rs452932, rs452384, rs31489, rs31490 for top, middle and lower panels, respectively. UMs: Cases of the discovery series, CTLs: Controls of the discovery series, ASW: African ancestry (Southwest USA), CEU: Utah residents with Northern and Western European ancestry from the CEPH collection, CHD: Chinese in Metropolitan Denver (Colorado), GIH: Gujarati Indians in Houston (Texas), JPT+CHB: Japanese in Tokyo (Japan) + Han Chinese in Beijing (China), LWK: Luhya in Webuye (Kenya), MKK: Maasai in Kinyawa (Kenya), TSI: Toscani (Italia). YRI: Yoruba in Ibadan (Nigeria). In bold, risk alleles of the haplotype.

**Supplementary Table 3.** Comparison of SNPs association between skin melanoma and our uveal melanoma GWAS.

| ***Skin melanoma GWAS*** | | | | | | ***Uveal melanoma association^1^*** | | | ***Uveal melanoma GWAS*** | | | |
| --- | --- | --- | --- | --- | --- | --- | --- | --- | --- | --- | --- | --- |
| **CHR** | **SNP** | **Gene** | **OR** | **95%CI** | ***P*-value** | **OR** | **95%CI** | ***P-value*** | **OR** | **95%CI** | ***P*-value** | **MAF**  **(UMs/CTLs)** |
| 5 | rs401681 | *TERT/CLPTM1L* | 0.88 | 0,82-0,95 | 1.0 x 10^-3^ | 1.17 | 0.98-1.41 | 8.6 x 10^-2^ | **1.78** | **1.06-2.96** | **1.3 x 10^-6^** | **0.45/0.41** |
| 5 | rs16891982 | *SLC45A2* | 0.42 | 0.35-0.50 | 1.5 x 10^-23^ | - | - | - | - | - | - |  |
| 6 | rs12203592 | *IRF4* | 1.55 | 1.25-1.92 | 5.8 x 10^-5^ | 1.47 | 1.18-1.82 | 6.3 x 10^-4^ | **1.88** | **1.03-3.44** | **2.5 x 10^-5^** | **0.23/0.13** |
| 9 | rs7023329 | *CDKN2A/MTAP* | 0.83 | 0.80-0.86 | 1.1 x 10^-25^ | 0.99 | 0.83-1.19 | 1 | 0.95 | 0.83-1.10 | 7.0 x 10^-1^ | 0.49/0.49 |
| 9 | rs3088440 | *CDKN2A* | 1.27 | 1.10-1.46 | 9.0 x 10^-4^ | - | - | - | - | - | - |  |
| 9 | rs1408799 | *TYRP1* | 0.91 | 0.84-0.98 | 1.2 x 10^-2^ | - | - | - | 0.95 | 0.82-1.11 | 6.8 x 10^-1^ | 0.33/0.34 |
| 11 | rs1393350 | *TYR* | 1.20 | 1.14-1.26 | 7.9 x 10^-12^ | 1.16 | 0.96-1.42 | 1.4 x 10^-1^ | 1.20 | 0.88-1.58 | 2.0 x 10^-1^ | 0.28/0.25 |
| 11 | rs1801516 | *ATM* | 0.84 | 0.79-0.89 | 1.5 x 10^-9^ | 0.82 | 0.62-1.08 | 1.8 x 10^-1^ | - | - | - |  |
| 11 | rs11263498 | *CCND1* | 1.11 | 1.05-1.18 | 4.6 x 10^-4^ | - | - | - | 1.14 | 0.89-1.48 | 2.5 x 10^-1^ | 0.42/0.39 |
| 12 | rs1544410 | *VDR* | 0.90 | 0.83-0.96 | 4.0 x 10^-3^ | - | - | - | 1.14 | 0.90-1.46 | 2.4 x 10^-1^ | 0.44/0.41 |
| 13 | rs17655 | *XPG* | 0.91 | 0.82-1.00 | 4.3 x 10^-2^ | - | - | - | - | - | - |  |
| 15 | rs12913832 | *HERC2/OCA2* | 0.69 | 0.61-0.79 | 4.3 x 10^-8^ | 0.53 | 0.42-0.67 | 8.5 x 10^-8^ | 0.63 | 0.40-1.01 | 2.9 x 10^-4^ | 0.29/0.39 |
| 15 | rs1129038 | *HERC2/OCA2* | 0.69 | 0.61-0.79 | 2.6 x 10^-8^ | 0.53 | 0.42-0.69 | 1.2 x 10^-7^ | - | - | - |  |
| 15 | rs916977 | *HERC2/OCA2* | - | - | 1.2 x 10^-43^ | 0.47 | 0.34-0.67 | 3.0 x 10^-7^ | **0.55** | **0.31-0.98** | **4.9 x 10^-5^** | **0.17/0.26** |
| 15 | rs4778138 | *HERC2/OCA2* | 0.80 | 0.69-0.92 | 6.9 x 10^-3^ | 0.58 | 0.42-0.80 | 5.1 x 10^-4^ | 0.72 | 0.47-1.12 | 3.5 x 10^-2^ | 0.15/0.19 |
| 15 | rs1800407 | *OCA2* | 1.38 | 1.09-1.70 | 7.0 x 10^-3^ | - | - | - | - | - | - |  |
| 16 | rs258322 | *CDK10* | 1.64 | 1.44-1.86 | 4.0 x 10^-14^ | - | - | - | 0.91 | 0.68-1.22 | 7.0 x 10^-1^ | 0.06/0.07 |
| 16 | rs4785763 | *AFG3L1* | 1.35 | 1.27-1.44 | 1.0 x 10^-20^ | - | - | - | 0.97 | 0.86-1.09 | 8.3 x 10^-1^ | 0.28/0.28 |
| 20 | rs4911442 | *NCAO6/ASIP* | 1.78 | 1.37-2.30 | 1.0 X 10^-4^ | - | - | - | 1.77 | 0.90-3.50 | 5.6 x 10^-3^ | 0.11/0.06 |
| 20 | rs1885120 | *MYH7B* | 1.55 | 1.41-1.71 | 1.6 x 10^-18^ | - | - | - | - | - | - |  |
| 21 | rs45430 | *MX2* | 0.89 | 0.85-0.94 | 1.1 x 10^-5^ | 0.96 | 0.80-1.16 | 7.1 x 10^-1^ | 0.74 | 0.81-1.11 | 6.4 x 10^-1^ | 0.39/0.41 |
| 22 | rs6001027 | *PLA2G6* | 0.86 | 0.80-0.95 | 7.5 x 10^-7^ | 1.07 | 0.88-1.29 | 5.0 x 10^-1^ | 1 | 0.97-1.03 | 9.8 x 10^-1^ | 0.39/0.39 |

CHR: Chromosome. OR: Odds ratio. 95%CI: Confidence interval at 95%, MAF: Minor Allele Frequency for uveal melanoma cases (UMs) and controls (CTLs). Data presented in the ‘Skin melanoma GWAS’ panel are from the meta-analysis by^2^, except for rs4601681^3^, for rs12203592^4^, for rs12913832 and rs1129038^5^, rs4778138^6^, rs916977^7^ and rs4911442^8^. Data presented in the ‘Uveal melanoma GWAS’ panel correspond to the results obtained by the current study. In bold, SNPs associated with OR under 10^-4^.

1. Ferguson R*, et al.* Genetic markers of pigmentation are novel risk loci for uveal melanoma. *Sci Rep* **6**, 31191 (2016).

2. Antonopoulou K*, et al.* Updated field synopsis and systematic meta-analyses of genetic association studies in cutaneous melanoma: the MelGene database. *J Invest Dermatol* **135**, 1074-1079 (2015).

3. Law MH*, et al.* Meta-analysis combining new and existing data sets confirms that the TERT-CLPTM1L locus influences melanoma risk. *J Invest Dermatol* **132**, 485-487 (2012).

4. Han J*, et al.* A germline variant in the interferon regulatory factor 4 gene as a novel skin cancer risk locus. *Cancer Res* **71**, 1533-1539 (2011).

5. Amos CI*, et al.* Genome-wide association study identifies novel loci predisposing to cutaneous melanoma. *Hum Mol Genet* **20**, 5012-5023 (2011).

6. Guedj M*, et al.* Variants of the MATP/SLC45A2 gene are protective for melanoma in the French population. *Hum Mutat* **29**, 1154-1160 (2008).

7. Kayser M*, et al.* Three genome-wide association studies and a linkage analysis identify HERC2 as a human iris color gene. *Am J Hum Genet* **82**, 411-423 (2008).

8. Maccioni L*, et al.* Variants at chromosome 20 (ASIP locus) and melanoma risk. *Int J Cancer* **132**, 42-54 (2013).

**Supplementary Table 4.** Combined risk analysis of rs11074306 (*OCA2* locus) and rs421284 (*CLPTM1L* locus).

Significance was measured using unconditional logistic regressions and the Cochran-Armitage test for trend.

| **Genotype rs11074306 (*OCA2*)*** | **NB** | **OR 95%CI rs421284 (*CLPTM1L*)** | ***P*-value** |
| --- | --- | --- | --- |
| AA | 133 | 2.3 1.18-2.86 | 1 x 10^-2^ |
| AG | 337 | 2.06 1.45-2.94 | 6 x 10^-5^ |
| GG | 190 | 1.8 1.22-4.44 | 6 x 10^-3^ |

*****G: risk allele for rs11074306. NB: Number of individual in the

association analysis; OR: Odds Ratio; 95% CI: Confidence

Interval at 95%.

**Supplementary Table 5.** Clinical characteristics of uveal melanoma cases included in the GWAS (discovery series).

| **Patient ID** | **Age** | **Sex** | **Location** | **Treatment** | **Statut** |
| --- | --- | --- | --- | --- | --- |
| B00G3UX | 74 | Female | Choroid | Enucleation | Metastases |
| B00G3UY | 48 | Female | Choroid | Enucleation | Metastases |
| B00G3UZ | 67 | Female | Choroid | Enucleation | Metastases |
| B00G3V0 | 64 | Male | Choroid | Enucleation | Metastases |
| B00G3V1 | 35 | Female | Choroid | Enucleation | Metastases |
| B00G3V2 | 71 | Male | Choroid | Enucleation | Metastases |
| B00G3V3 | 56 | Female | Choroid | Enucleation | Disease-free |
| B00G3V4 | 76 | Male | Choroid | Enucleation | Metastases |
| B00G3V5 | 31 | Male | Choroid | Enucleation | Disease-free |
| B00G3V6 | 69 | Male | Choroid | Enucleation | Metastases |
| B00G3V7 | 52 | Female | Choroid | Enucleation | Disease-free |
| B00G3V8 | 55 | Female | Choroid | Enucleation | Metastases |
| B00G3VD | 61 | Male | Choroid | Protontherapy | Metastases |
| B00G3VH | 73 | Male | Choroid | Enucleation | Metastases |
| B00G3VI | 57 | Female | Choroid | Protontherapy | Metastases |
| B00G3VL | 61 | Female | Choroid | Protontherapy | Metastases |
| B00G3VN | 66 | Male | Choroid | Enucleation | Metastases |
| B00G3VO | 58 | Female | Choroid | Protontherapy | Metastases |
| B00G3VS | 58 | Male | Choroid | Protontherapy | Metastases |
| B00G3VT | 24 | Female | Choroid | Enucleation | Metastases |
| B00G3VU | 28 | Female | Choroid | Protontherapy | Metastases |
| B00G3VV | 35 | Male | Choroid | Protontherapy | Metastases |
| B00G3VX | 37 | Male | Choroid | Enucleation | Metastases |
| B00G3VZ | 74 | Female | Choroid | Enucleation | Metastases |
| B00G3W2 | 69 | Male | Choroid | Protontherapy | Metastases |
| B00G3W3 | 40 | Female | Choroid | Protontherapy | Disease-free |
| B00G3W4 | 49 | Female | Choroid | Protontherapy | Disease-free |
| B00G3W5 | 52 | Female | Choroid | Protontherapy | Disease-free |
| B00G3W7 | 75 | Male | Choroid | Protontherapy | Disease-free |
| B00G3W8 | 84 | Female | Choroid | Protontherapy | Disease-free |
| B00G3W9 | 41 | Female | Choroid | Protontherapy | Disease-free |
| B00G3WA | 71 | Female | Choroid | Protontherapy | Disease-free |
| B00G3WB | 46 | Female | Choroid | Protontherapy | Disease-free |
| B00G3WC | 54 | Male | Choroid | Protontherapy | Metastases |
| B00G3WD | 63 | Male | Choroid | Protontherapy | Metastases |
| B00G3WH | 57 | Female | Choroid | Enucleation | Metastases |
| B00G3WJ | 63 | Male | Choroid | Protontherapy | Metastases |
| B00G3WK | 60 | Female | Choroid | Brachytherapy | Disease-free |
| B00G3WL | 67 | Female | Choroid | Protontherapy | Metastases |
| B00G3WM | 67 | Female | Choroid | Protontherapy | Disease-free |
| B00G3WO | 72 | Male | Choroid | Protontherapy | Disease-free |
| B00G3WP | 56 | Male | Choroid | Protontherapy | Disease-free |
| B00G3WQ | 58 | Female | Choroid | Protontherapy | Disease-free |
| B00G3WS | 80 | Female | Choroid | Protontherapy | Metastases |
| B00G3WT | 62 | Male | Choroid | Protontherapy | Disease-free |
| B00G3WU | 84 | Male | Choroid | Protontherapy | Disease-free |
| B00G3WV | 74 | Female | Choroid | Protontherapy | Disease-free |
| B00G3WW | 68 | Female | Choroid | Brachytherapy | Disease-free |
| B00G3WX | 82 | Female | Choroid | Protontherapy | Disease-free |
| B00G3WY | 62 | Female | Choroid | Protontherapy | Disease-free |
| B00G3WZ | 43 | Male | Choroid | Protontherapy | Metastases |
| B00G3X0 | 71 | Male | Choroid | Protontherapy | Metastases |
| B00G3X1 | 85 | Male | Choroid | Protontherapy | Disease-free |
| B00G3X2 | 42 | Female | Choroid | Protontherapy | Disease-free |
| B00G3X6 | 53 | Male | Choroid | Protontherapy | Disease-free |
| B00G3XB | 19 | Female | Choroid | Brachytherapy | Disease-free |
| B00G3XC | 43 | Female | Choroid | Protontherapy | Disease-free |
| B00G3XD | 55 | Male | Choroid | Enucleation | Metastases |
| B00G3XE | 61 | Male | Choroid | Enucleation | Disease-free |
| B00G3XF | 57 | Male | Choroid | Brachytherapy | Disease-free |
| B00G3XL | 58 | Male | Choroid | Enucleation | Metastases |
| B00G3XM | 62 | Male | Choroid | Protontherapy | Metastases |
| B00G3XN | 74 | Female | Choroid | Protontherapy | Metastases |
| B00G3XO | 51 | Male | Choroid | Enucleation | Metastases |
| B00G3XP | 68 | Male | Choroid | Protontherapy | Metastases |
| B00G3XR | 48 | Female | Choroid | Protontherapy | Metastases |
| B00G3XS | 64 | Male | Choroid | Enucleation | Metastases |
| B00G3XT | 57 | Male | Choroid | Brachytherapy | Metastases |
| B00G3XU | 35 | Female | Choroid | Enucleation | Metastases |
| B00G3XV | 41 | Male | Choroid | Protontherapy | Metastases |
| B00G3XW | 73 | Female | Choroid | Enucleation | Metastases |
| B00G3XX | 60 | Female | Choroid | Enucleation | Metastases |
| B00G3XY | 41 | Male | Choroid | Protontherapy | Disease-free |
| B00G3Y0 | 38 | Female | Choroid | Protontherapy | Disease-free |
| B00G3Y1 | 74 | Female | Choroid | Protontherapy | Disease-free |
| B00G3Y2 | 80 | Male | Choroid | Enucleation | Metastases |
| B00G3Y3 | 52 | Male | Choroid | Protontherapy | Disease-free |
| B00G3Y8 | 52 | Female | Choroid | Protontherapy | Disease-free |
| B00G3YC | 36 | Male | Choroid | Protontherapy | Metastases |
| B00G3YE | 59 | Male | Choroid | Protontherapy | Disease-free |
| B00G3YF | 28 | Male | Choroid | Protontherapy | Disease-free |
| B00G3YG | 62 | Female | Choroid | Protontherapy | Disease-free |
| B00G3YH | 65 | Female | Choroid | Protontherapy | Disease-free |
| B00G3YI | 65 | Male | Choroid | Protontherapy | Disease-free |
| B00G3YJ | 63 | Female | Choroid | Protontherapy | Disease-free |
| B00G3YK | 66 | Female | Choroid | Protontherapy | Disease-free |
| B00G3YL | 62 | Male | Choroid | Protontherapy | Disease-free |
| B00G3YM | 79 | Male | Choroid | Protontherapy | Metastases |
| B00G3YN | 58 | Male | Choroid | Enucleation | Disease-free |
| B00G3YO | 83 | Female | Choroid | Enucleation | Metastases |
| B00G3YP | 41 | Male | Choroid | Protontherapy | Disease-free |
| B00G3YQ | 58 | Male | Choroid | Protontherapy | Disease-free |
| B00G3YR | 72 | Female | Choroid | Protontherapy | Disease-free |
| B00G3YS | 63 | Male | Choroid | Enucleation | Disease-free |
| B00G3YT | 62 | Male | Choroid | Protontherapy | Disease-free |
| B00G3YU | 52 | Male | Choroid | Enucleation | Metastases |
| B00G3YV | 69 | Male | Choroid | Protontherapy | Disease-free |
| B00G3YW | 56 | Male | Choroid | Protontherapy | Disease-free |
| B00G3YX | 45 | Male | Choroid | Protontherapy | Disease-free |
| B00G3YY | 63 | Male | Choroid | Protontherapy | Disease-free |
| B00G3YZ | 74 | Male | Choroid | Protontherapy | Disease-free |
| B00G3Z5 | 64 | Female | Choroid | Protontherapy | Disease-free |
| B00G3Z6 | 56 | Male | Choroid | Protontherapy | Metastases |
| B00G3Z7 | 79 | Female | Choroid | Enucleation | Disease-free |
| B00G3ZA | 79 | Male | Choroid | Enucleation | Disease-free |
| B00G3ZB | 59 | Male | Choroid | Enucleation | Disease-free |
| B00G3ZC | 58 | Female | Choroid | Protontherapy | Metastases |
| B00G3ZD | 76 | Female | Choroid | Enucleation | Metastases |
| B00G3ZE | 71 | Male | Choroid | Enucleation | Disease-free |
| B00G3ZF | 26 | Male | Choroid | Protontherapy | Disease-free |
| B00G3ZH | 75 | Male | Choroid | Enucleation | Disease-free |
| B00G3ZI | 76 | Female | Choroid | Protontherapy | Disease-free |
| B00G3ZJ | 42 | Male | Choroid | Protontherapy | Disease-free |
| B00G3ZK | 64 | Male | Choroid | Enucleation | Metastases |
| B00G3ZL | 43 | Female | Choroid | Enucleation | Metastases |
| B00G3ZN | 70 | Male | Choroid | Protontherapy | Disease-free |
| B00G3ZO | 79 | Female | Choroid | Protontherapy | Disease-free |
| B00G3ZP | 84 | Female | Choroid | Protontherapy | Disease-free |
| B00G3ZQ | 51 | Male | Choroid | Protontherapy | Disease-free |
| B00G3ZR | 82 | Male | Choroid | Protontherapy | Disease-free |
| B00G3ZS | 56 | Male | Choroid | Protontherapy | Disease-free |
| B00G3ZT | 31 | Female | Choroid | Enucleation | Disease-free |
| B00G3ZU | 77 | Female | Choroid | Enucleation | Metastases |
| B00G3ZX | 40 | Female | Choroid | Protontherapy | Disease-free |
| B00G3ZY | 44 | Female | Choroid | Protontherapy | Disease-free |
| B00G3ZZ | 70 | Male | Choroid | Enucleation | Metastases |
| B00G400 | 76 | Female | Choroid | Protontherapy | Disease-free |
| B00G402 | 66 | Male | Choroid | Protontherapy | Disease-free |
| B00G403 | 29 | Male | Choroid | Protontherapy | Disease-free |
| B00G404 | 77 | Male | Choroid | Protontherapy | Disease-free |
| B00G405 | 78 | Male | Choroid | Protontherapy | Disease-free |
| B00G406 | 66 | Female | Choroid | Enucleation | Disease-free |
| B00G408 | 72 | Male | Choroid | Protontherapy | Disease-free |
| B00G409 | 59 | Male | Choroid | Protontherapy | Disease-free |
| B00G40A | 64 | Female | Ciliary bodies | Brachytherapy | Disease-free |
| B00G40B | 35 | Male | Choroid | Protontherapy | Disease-free |
| B00G40C | 52 | Male | Choroid | Enucleation | Disease-free |
| B00G40D | 78 | Male | Choroid | Protontherapy | Disease-free |
| B00G40E | 72 | Male | Choroid | Protontherapy | Disease-free |
| B00G40F | 62 | Male | Choroid | Protontherapy | Disease-free |
| B00G40G | 84 | Female | Choroid | Enucleation | Disease-free |
| B00G40I | 55 | Female | Choroid | Enucleation | Disease-free |
| B00G40J | 60 | Female | Choroid | Protontherapy | Disease-free |
| B00G40K | 85 | Female | Choroid | Protontherapy | Disease-free |
| B00G40L | 53 | Female | Choroid | Protontherapy | Disease-free |
| B00G40O | 75 | Female | Choroid | Enucleation | Metastases |
| B00G40P | 48 | Female | Choroid | Enucleation | Metastases |
| B00G40Q | 55 | Male | Choroid | Protontherapy | Disease-free |
| B00G40R | 66 | Male | Choroid | Protontherapy | Disease-free |
| B00G40S | 77 | Female | Choroid | Protontherapy | Disease-free |
| B00G40T | 25 | Female | Choroid | Brachytherapy | Disease-free |
| B00G40U | 83 | Female | Choroid | Protontherapy | Disease-free |
| B00G40V | 84 | Female | Choroid | Protontherapy | Disease-free |
| B00G40W | 72 | Female | Choroid | Protontherapy | Disease-free |
| B00G40X | 37 | Male | Choroid | Protontherapy | Disease-free |
| B00G40Y | 82 | Female | Choroid | Protontherapy | Disease-free |
| B00G410 | 77 | Male | Choroid | Protontherapy | Disease-free |
| B00G411 | 84 | Male | Choroid | Protontherapy | Disease-free |
| B00G412 | 65 | Female | Choroid | Protontherapy | Disease-free |
| B00G413 | 48 | Male | Choroid | Protontherapy | Disease-free |
| B00G414 | 65 | Male | Choroid | Protontherapy | Disease-free |
| B00G415 | 69 | Female | Choroid | Protontherapy | Disease-free |
| B00G416 | 61 | Female | Choroid | Protontherapy | Disease-free |
| B00G417 | 36 | Male | Choroid | Enucleation | Disease-free |
| B00G418 | 73 | Male | Choroid | Protontherapy | Metastases |
| B00G419 | 70 | Male | Choroid | Enucleation | Disease-free |
| B00G41A | 77 | Female | Choroid | Protontherapy | Disease-free |
| B00G41B | 53 | Female | Choroid | Enucleation | Disease-free |
| B00G41D | 78 | Male | Choroid | Protontherapy | Disease-free |
| B00G41E | 85 | Male | Choroid | Protontherapy | Disease-free |
| B00G41F | 44 | Female | Choroid | Protontherapy | Disease-free |
| B00G41G | 59 | Female | Choroid | Protontherapy | Disease-free |
| B00G41H | 54 | Male | Choroid | Protontherapy | Disease-free |
| B00G41J | 52 | Female | Choroid | Protontherapy | Disease-free |
| B00G41K | 79 | Male | Choroid | Protontherapy | Disease-free |
| B00G41L | 52 | Male | Choroid | Protontherapy | Disease-free |
| B00G41M | 53 | Female | Choroid | Protontherapy | Disease-free |
| B00G82J | 63 | Female | Choroid | Protontherapy | Disease-free |
| B00G82K | 38 | Male | Choroid | Protontherapy | Disease-free |
| B00G82L | 65 | Male | Choroid | Protontherapy | Disease-free |
| B00G82M | 77 | Female | Choroid | Protontherapy | Disease-free |
| B00G82N | 63 | Male | Choroid | Protontherapy | Disease-free |
| B00G82O | 77 | Male | Choroid | Protontherapy | Disease-free |
| B00G82P | 44 | Male | Choroid | Brachytherapy | Disease-free |
| B00G82Q | 62 | Male | Choroid | Enucleation | Disease-free |
| B00G82S | 71 | Female | Choroid | Protontherapy | Disease-free |
| B00G82T | 43 | Male | Choroid | Protontherapy | Disease-free |
| B00G82U | 61 | Male | Choroid | Enucleation | Disease-free |
| B00G82W | 64 | Female | Choroid | Protontherapy | Disease-free |
| B00G82X | 78 | Male | Choroid | Protontherapy | Disease-free |
| B00G82Y | 67 | Male | Choroid | Enucleation | Metastases |
| B00G82Z | 75 | Female | Choroid | Protontherapy | Disease-free |
| B00G830 | 74 | Male | Choroid | Protontherapy | Disease-free |
| B00G831 | 83 | Female | Choroid | Protontherapy | Disease-free |
| B00G832 | 54 | Male | Choroid | Protontherapy | Disease-free |
| B00G833 | 60 | Male | Choroid | Protontherapy | Disease-free |
| B00G834 | 94 | Male | Choroid | Protontherapy | Disease-free |
| B00G835 | 82 | Female | Choroid | Brachytherapy | Disease-free |
| B00G836 | 44 | Male | Choroid | Brachytherapy | Disease-free |
| B00G837 | 61 | Female | Choroid | Protontherapy | Disease-free |
| B00G838 | 83 | Female | Choroid | Protontherapy | Disease-free |
| B00G839 | 49 | Female | Choroid | Enucleation | Disease-free |
| B00G83A | 51 | Female | Choroid | Enucleation | Disease-free |
| B00G83B | 39 | Female | Choroid | Protontherapy | Disease-free |
| B00G83C | 72 | Male | Choroid | Protontherapy | Metastases |
| B00G83D | 69 | Male | Choroid | Protontherapy | Disease-free |
| B00G83E | 66 | Male | Choroid | Protontherapy | Disease-free |
| B00G83G | 83 | Female | Choroid | Enucleation | Disease-free |
| B00G83H | 49 | Female | Choroid | Protontherapy | Disease-free |
| B00G83I | 70 | Male | Choroid | Protontherapy | Disease-free |
| B00G83J | 48 | Female | Choroid | Protontherapy | Disease-free |
| B00G83K | 72 | Female | Choroid | Protontherapy | Disease-free |
| B00G83L | 61 | Female | Choroid | Enucleation | Disease-free |
| B00G83M | 74 | Female | Choroid | Enucleation | Metastases |
| B00G83N | 64 | Female | Choroid | Brachytherapy | Disease-free |
| B00G83O | 66 | Female | Choroid | Protontherapy | Disease-free |
| B00G83Q | 63 | Female | Choroid | Protontherapy | Disease-free |
| B00G83R | 60 | Female | Choroid | Protontherapy | Disease-free |
| B00G83S | 29 | Female | Choroid | Protontherapy | Disease-free |
| B00G83T | 51 | Male | Choroid | Protontherapy | Disease-free |
| B00G83U | 50 | Female | Choroid | Protontherapy | Disease-free |
| B00G83V | 61 | Female | Choroid | Brachytherapy | Disease-free |
| B00G83W | 68 | Male | Choroid | Protontherapy | Disease-free |
| B00GGU6 | 46 | Male | Choroid | Protontherapy | Disease-free |
| B00GGU7 | 60 | Male | Choroid | Protontherapy | Disease-free |
| B00GGU8 | 62 | Male | Choroid | Enucleation | Disease-free |
| B00GGUA | 31 | Male | Choroid | Protontherapy | Disease-free |
| B00GGUB | 55 | Male | Choroid | Enucleation | Disease-free |
| B00GGUC | 70 | Male | Choroid | Protontherapy | Disease-free |
| B00GGUD | 71 | Female | Choroid | Protontherapy | Disease-free |
| B00GGUE | 36 | Male | Choroid | Protontherapy | Disease-free |
| B00GGUF | 71 | Male | Choroid | Enucleation | Disease-free |
| B00GGUG | 52 | Female | Choroid | Protontherapy | Disease-free |
| B00GGUH | 43 | Male | Choroid | Protontherapy | Disease-free |
| B00GGUI | 46 | Male | Choroid | Protontherapy | Disease-free |
| B00GGUJ | 75 | Male | Choroid | Protontherapy | Disease-free |
| B00GGUK | 77 | Male | Choroid | Protontherapy | Disease-free |
| B00GGUL | 86 | Female | Choroid | Protontherapy | Disease-free |
| B00GGUM | 50 | Male | Choroid | Protontherapy | Disease-free |
| B00GGUN | 24 | Female | Choroid | Protontherapy | Disease-free |
| B00GGUO | 54 | Male | Iris | Protontherapy | Disease-free |
| B00GGUP | 84 | Female | Choroid | Protontherapy | Disease-free |
| B00GGUQ | 75 | Male | Choroid | Protontherapy | Disease-free |
| B00GGUR | 46 | Female | Choroid | Protontherapy | Disease-free |
| B00GGUS | 73 | Female | Choroid | Enucleation | Metastases |
| B00GGUT | 79 | Male | Choroid | Protontherapy | Disease-free |
| B00GGUU | 61 | Female | Choroid | Enucleation | Disease-free |
| B00GGUV | 88 | Female | Choroid | Enucleation | Metastases |
| B00GGUW | 69 | Male | Choroid | Enucleation | Metastases |
| B00GGUX | 72 | Female | Choroid | Protontherapy | Disease-free |
| B00GGUY | 65 | Female | Choroid | Enucleation | Disease-free |
| B00GGUZ | 59 | Female | Choroid | Protontherapy | Disease-free |
| B00GGV1 | 49 | Female | Choroid | Protontherapy | Disease-free |
| B00GGV2 | 82 | Female | Choroid | Enucleation | Disease-free |
| B00GGV3 | 62 | Female | Choroid | Protontherapy | Disease-free |
| B00GGV4 | 81 | Female | Choroid | Protontherapy | Disease-free |
| B00GGV5 | 58 | Male | Choroid | Enucleation | Disease-free |
| B00GGV6 | 60 | Female | Choroid | Protontherapy | Disease-free |
| B00GGV7 | 47 | Female | Choroid | Enucleation | Disease-free |

**Supplementary Table 6.** TaqMan® assays used in the validation study.

| **Assay ID/Name** | **Gene ID or REF** | **Comments** |
| --- | --- | --- |
| C_2396811_10 | rs421284 | Chr.5: 1325590 |
| C_8769675_20 | rs452932 | Chr.5: 1330253 |
